# Palonosetron versus ondansetron for prevention of nausea and vomiting after total abdominal hysterectomy under spinal anesthesia with intrathecal morphine: a double-blind, randomized controlled trial

**DOI:** 10.1186/s12871-019-0830-7

**Published:** 2019-08-17

**Authors:** Guilherme Oliveira Campos, Marcelo de Jesus Martins, Gabriel Nascimento Jesus, Paulo Roberto Rios de Oliveira, Caio Nogueira Lessa, João Carlos Macêdo Fernandes de Oliveira Junior, Lucas Jorge Santana de Castro Alves, Rodrigo Leal Alves, Norma Sueli Pinheiro Módolo

**Affiliations:** 1 Department of Anesthesiology, São Rafael Hospital, D’Or Institute for Research and Education (IDOR), Salvador, Brazil; 2 Department of Anesthesiology, Cardiopulmonar Hospital, Salvador, Brazil; 3 Department of Anesthesiology, São Paulo State University, Botucatu, Brazil; 4 Federal University of Bahia, Salvador, Brazil; 5 Department of Gynecology, São Rafael Hospital, D’Or Institute for Research and Education (IDOR), Salvador, Brazil; 6 Department of Anesthesiology, Santo Antonio Hospital, Salvador, Brazil

**Keywords:** Antiemetics, Morphine, Ondansetron, Postoperative nausea and vomiting, Spinal anesthesia

## Abstract

**Background:**

Hysterectomy is a widely performed surgery and neuraxial anesthesia with intrathecal morphine provides superior quality of recovery. Postoperative nausea and vomiting (PONV) is a frequent problem with intrathecal morphine use. Although palonosetron is effective for prevention of PONV after general anesthesia, its efficacy after neuraxial anesthesia has not been established. This study was conducted to compare the use of palonosetron with ondansetron for PONV prophylaxis in patients at a high risk of PONV during total abdominal hysterectomy (TAH) under spinal anesthesia with intrathecal morphine.

**Methods:**

This prospective, randomized double-blind study conducted at São Rafael Hospital involved 140 American Society of Anesthesiologists physical status I or II women who underwent TAH under spinal anesthesia with intrathecal morphine and who had at least 3 risk factors for PONV based on Apfel’s simplified score. The patients were randomized into two groups: one received palonosetron whereas the other received ondansetron. All patients received spinal anesthesia with intrathecal morphine, as well as dexamethasone plus palonosetron or ondansetron for PONV prophylaxis. The overall incidence of PONV, incidence of early- and late-onset nausea and vomiting, severity of nausea, and use of rescue antiemetics were recorded.

**Results:**

The overall incidence of PONV was 42.9% in the palonosetron group and 52.9% in the ondansetron group (*p* > 0.05). No significant differences existed in the incidence of early- and late-onset nausea or early-onset vomiting between the two groups. The incidence of late-onset vomiting was significantly lower in the palonosetron group.

**Conclusions:**

Palonosetron exhibited efficacy similar to that of ondansetron for reducing the overall incidence of PONV after TAH under spinal anesthesia with intrathecal morphine; however, palonosetron reduced the incidence of late-onset vomiting significantly better than ondansetron.

**Trial registration:**

RBR-4gnm8n (ensaiosclinicos.gov.br), date of registration: August 18, 2014.

## Background

Postoperative nausea and vomiting (PONV) is a common perioperative complication that is associated with clinical and economic consequences, including wound dehiscence, delayed nutrition, prolonged hospital stay, and reduced patient satisfaction. Despite advances in prevention and treatment, the incidence of PONV remains high, especially in high-risk patients [1].

Total abdominal hysterectomy (TAH) is a surgery that is often performed worldwide, and neuraxial anesthesia with intrathecal morphine has been shown to provide improved quality of recovery because of superior, prolonged pain control after TAH [2]. However, PONV is a frequent complication after TAH due to both the effects of intrathecal morphine and the intrinsic characteristics of the patients [3–7].

Palonosetron is a second generation 5HT-3 receptor antagonist that has a greater binding affinity and a longer plasma half-life than other drugs in the same class [8, 9]. Studies have reported the superiority of palonosetron relative to other 5HT-3 receptor antagonists for the prevention of PONV in patients undergoing general anesthesia. However, little is known regarding the ability of palonosetron to prevent PONV after spinal anesthesia [10–12].

The aim of the current study was to compare the efficacy of palonosetron versus ondansetron, both in combination with dexamethasone, for PONV prophylaxis in patients undergoing TAH under spinal anesthesia with intrathecal morphine.

## Methods

This study was approved by the Research Ethics Committee (1.238.882/2015) of São Rafael Hospital, a tertiary care hospital in Salvador, Brazil. The trial was conducted from January to October of 2015, in adherence with the Consolidated Standards of Reporting Trials (CONSORT) guidelines, and was registered with the Brazilian Clinical Trial Registry (RBR-4gnm8n, August 18, 2014).

After they had provided written informed consent, 175 consecutive female patients scheduled to undergo elective TAH were recruited for this prospective, randomized, double-blinded study. The inclusion criteria were age 18 to 65 years, American Society of Anesthesiologists physical status I or II, and at least 3 risk factors for PONV as determined by Apfel’s simplified risk score. Exclusion criteria were body mass index > 35, contraindications to spinal anesthesia, use of corticosteroids or antiemetic medications in the 24 h preceding the surgery, and allergy to any medication used in the study protocol.

After enrollment, patients were randomly assigned in a 1:1 ratio to receive either ondansetron or palonosetron. The group assignment was presented in opaque sealed envelopes to a pharmacist who was not involved in the study. Randomization was performed with a block size of 6 using a central web-based system. A nurse who was not involved in the study opened the envelopes and prepared the medications as injectable solutions of either palonosetron 0.075 mg or ondansetron 4 mg, both diluted in normal saline to a total volume of 2 mL, in identical syringes. The study drug was administered intravenously (i.v.) immediately after the spinal block.

Preoperative fasting was initiated at midnight for all patients, and none received premedication. After arrival in the operating room, standard monitoring was applied (electrocardiography, pulse oximetry, and a non-invasive blood pressure cuff) and the subjects received 10 mL/kg of lactated Ringer’s solution i.v., 2 mg midazolam i.v., and 50 μg fentanyl i.v.

The patients were then placed in a sitting position, and a spinal block was performed at the L3–L4 or L4–L5 interspace using a 27-gauge Sprotte needle, followed by intrathecal injection of 15 mg hyperbaric bupivacaine and 100 μg preservative-free morphine. An appropriate block level (T6 dermatome) was confirmed prior to skin incision. Patients in both groups received additional titrated doses of midazolam (up to 10 mg i.v.) to maintain a level of sedation between − 1 and − 3 on the Richmond Agitation-Sedation Scale (0 for alert and calm, − 5 for unarousable). Ephedrine 5 mg i.v. was titrated to maintain an arterial blood pressure within 20% of baseline. Atropine 0.5 mg i.v. was given as needed to maintain a heart rate above 50 beats per minute.

Considering the high risk of PONV and ethical issues, all patients received dexamethasone 8 mg i.v. just after placement of the i.v. catheter as part of the PONV prophylaxis regimen. For postoperative analgesia, all patients received a multimodal regimen consisting of ketoprofen 100 mg i.v. every 8 h and metamizole 2 g i.v. every 6 h, beginning in the operating room. Morphine 3 mg i.v. up to every 4 h was used if the patient’s pain score was greater than 4 on the visual analog scale (VAS; 0 = no pain, 10 = the worst pain imaginable). Patients were observed in the post-anesthesia care unit for at least 1 h after surgery before they were transferred to the ward.

The incidence of PONV, severity of nausea, and the use of rescue antiemetics were evaluated at 1, 6, 24, and 48 h after surgery. An episode of vomiting was defined as either vomiting (expulsion of stomach contents) or retching (involuntary attempt to vomit that did not expel stomach contents). Severity of nausea was assessed using a VAS ranging from 0 to 10 (0 = no nausea, 10 = worst nausea imaginable). Rescue antiemetics (metoclopramide 10 mg i.v. followed by ondansetron 4 mg i.v. if there was no response to metoclopramide) were administered upon the patient’s request or with the onset of vomiting.

A simplified PONV impact scale questionnaire consisting of two questions was administered to all patients just before hospital discharge. A score from 0 to 6 was derived based the patient’s answers to the questions and scores above 4 were considered to indicate clinically significant PONV [13]. Patients were also asked to rate their experience regarding the management of PONV on a 4-point scale (poor, average, good, or excellent). Adverse events, such as dizziness and headache, were also investigated and recorded during the 48-h observation period.

The primary outcome measured was the incidence of PONV during the entire observation period. Secondary outcomes included the incidence of either nausea or vomiting, severity of nausea, use of rescue antiemetics, incidence of clinically important PONV, and overall patient satisfaction with the management of PONV. Early-onset nausea and/or vomiting were considered to occur within the first 6 h after surgery, whereas late-onset nausea and/or vomiting were considered to occur between 6 and 48 h after surgery.

In order to calculate the sample size, we conducted a retrospective review of the incidence of PONV in patients who had undergone TAH in the preceding year at São Rafael Hospital. A PONV incidence rate of approximately 60% was observed in patients who received ondansetron and dexamethasone in the operating room. It was estimated that a sample size of 67 patients per group would achieve 80% power for the detection of a 40% reduction in the incidence of PONV in the palonosetron group with an alpha error of 5% [14]. To account for patient dropout, we randomized 70 patients to each group.

The analysis was performed on an intention-to-treat basis using data from all randomized patients who underwent TAH. Dichotomous variables are expressed as relative and absolute frequencies. As the continuous variables in the study did not yield normal distributions, they are expressed as medians and interquartile ranges, as were the ordinal variables. The non-parametric Mann-Whitney test was used to compare data derived from continuous and ordinal variables in the two groups. Dichotomous variables were analyzed using the *χ*^2^ test or Fisher’s exact test as appropriate, and *P* < 0.05 was considered statistically significant. Statistical analysis was performed using SPSS for Windows (version 14, SPSS Inc., Chicago, IL, USA).

## Results

**Fig. 1:**
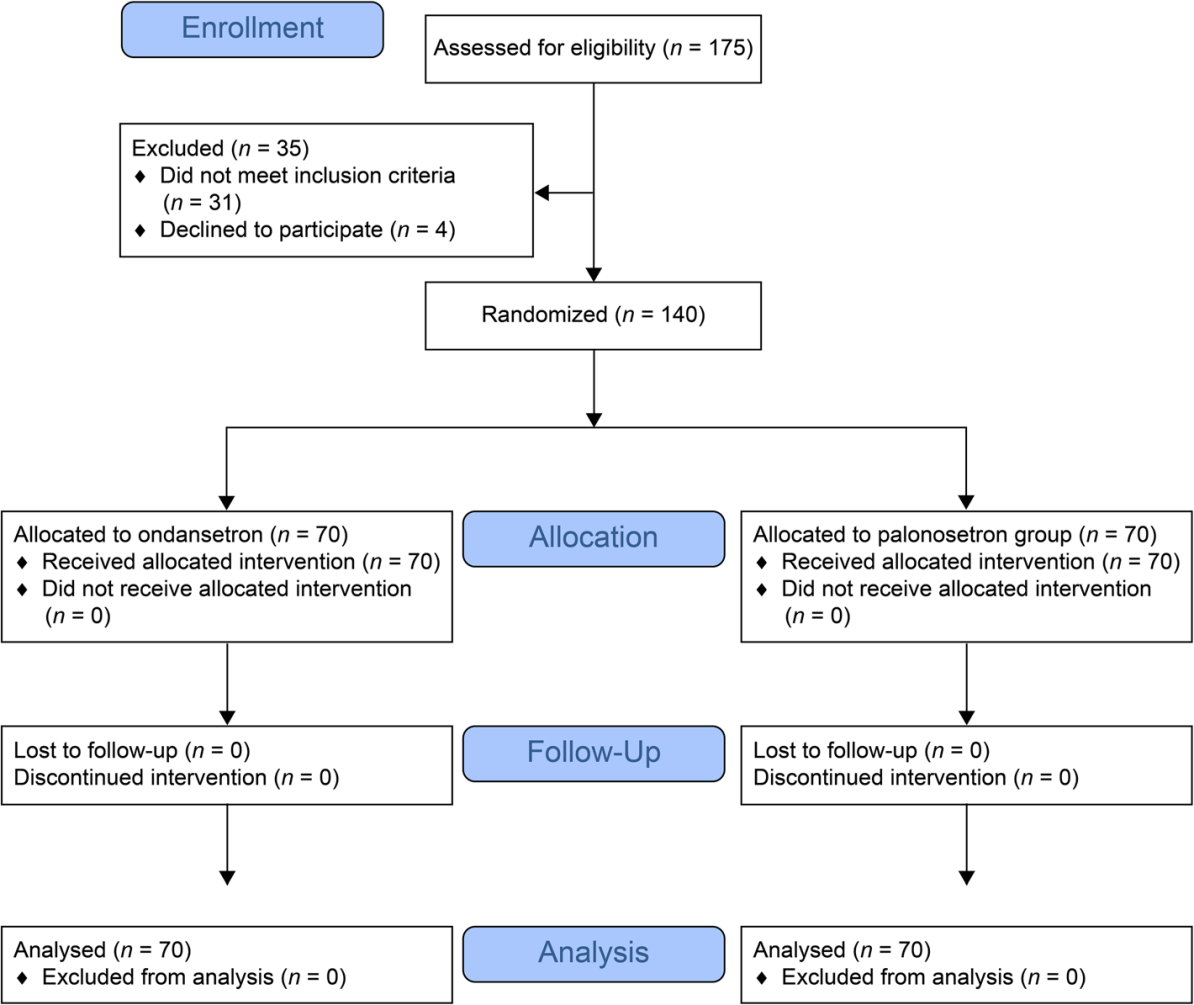
CONSORT flow diagram

Of the 175 patients who were initially screened, 140 met the inclusion and exclusion criteria and were randomly assigned to a study group. All patients who underwent randomization completed the trial, and there were 70 patients in each group (Fig. 1). The groups were well matched for age, height, body mass index, clinical comorbidities, smoking status, and preoperative fasting time. There were no significant differences in the duration of anesthesia, volume of infused crystalloid, total dose of midazolam, or use of vasopressors or atropine (Table 1).

**Table 1:** Characteristics, anesthetic, and surgical data of patients in the ondansetron and palonosetron groups

	Ondansetron	Palonosetron	*P* value
Age (years)	44 (41/47)	45 (42/48)	0.334
Weight (kg)	68.4 ± 10.7	70.3 ± 12.0	0.317
Height (cm)	158 ± 6	159 ± 5	0.609
Body mass index (kg/m^2^)	27.2 ± 3.9	27.7 ± 4.0	0.453
Motion sickness/previous PONV	7.1%	5.7%	0.730
Smoking	0%	1.4%	1.000
Hypertension	22.8%	27.1%	0.558
Diabetes mellitus	4.2%	1.4%	0.620
Other comorbidities	15.7%	8.5%	0.301
Fasting time (minutes)	770 (660/900)	720 (600/900)	0.508
Use of atropine	1.4%	10.0%	0.063
Use of ephedrine	20.0%	25.7%	0.421
Midazolam total dose (mg)	4.87 ± 1.8	4.8 ± 2.0	0.750
Duration of anesthesia (minutes)	95 (80/111)	90 (80/105)	0.242
Time in post-anesthesia care unit (minutes)	60 (55/66)	60 (55/65)	0.779

Values are presented as median (1st and 3rd quartiles), mean ± SD, or relative frequency (%)

*PONV* postoperative nausea and vomiting

There were no significant differences in the incidence of PONV during the total, early, or late periods between the two groups. There was also no significant difference in the severity of nausea between the two groups. There was a significantly lower incidence of late-onset vomiting in the palonosetron group than in the ondansetron group; however, there was no significant difference in the overall incidence of vomiting or in the incidence of early-onset vomiting. The need for rescue medication for PONV and the total dose of metoclopramide was similar between the two groups (Table 2).

**Table 2:** Frequencies of PONV and use of rescue medication in the ondansetron and palonosetron groups

	Ondansetron	Palonosetron	*P* value
PONV	52.9%	42.9%	0.236
Nausea	51.4%	42.9%	0.310
Early-onset nausea	27.1%	21.4%	0.430
Late-onset nausea	35.7%	30.0%	0.472
Vomiting	35.7%	22.9%	0.095
Early-onset vomiting	20.0%	14.3%	0.370
Late vomiting^*^	27.1%	11.4%	0.018
Use of rescue medication	30.0%	27.1%	0.708
Cumulative dose of metoclopramide (mg)^*^	0 (0/10)	0 (0/10)	0.840

Early-onset regarded as ≤6 h after surgery and late-onset regarded as 6–48 h after surgery

*PONV* postoperative nausea and vomiting

^#^Presented as median (1st and 3rd quartiles)

^*^P < 0.05

There were no significant differences in patient satisfaction with the management of PONV between the two groups (Table 3). The incidence of clinically significant PONV was low in both groups, as determined by the simplified PONV impact scale; however, there was no statistically significant difference in the incidence of clinically significant PONV between the two groups (Table 3) [13].

**Table 3:** Frequency of moderate/severe nausea, clinically significant PONV, and low patient satisfaction with PONV control

	Ondansetron	Palonosetron	*P* value
Moderate/severe nausea (VAS ≥ 5)	44.2%	35.7%	0.301
Clinically significant PONV (^a^score ≥ 5)	5.7%	2.8%	0.681
Low patient satisfaction with PONV control	15.7%	10.0%	0.313

*PONV* postoperative nausea and vomiting, *VAS* visual analog scale

^a^Simplified PONV impact scale [13]

The degree of postoperative pain, the need for rescue i.v. morphine, and the cumulative consumption of rescue i.v. morphine were similar between the two groups (Table 4). No patient had symptoms suggestive of postdural puncture headache. The incidence of adverse effects potentially attributable to serotonin receptor antagonists, such as dizziness and headache, was low in both groups (no statistically significant difference was detected), and adverse effects were considered mild in all cases (data not shown).

**Table 4:** Severity of pain, frequency of rescue analgesic use, and cumulative morphine consumption

	Ondansetron	Palonosetron	*P* value
Severity of pain (VAS)^a^	4 (0/7)	5 (0/7)	0.377
Use of morphine	5.7%	8.6%	0.745
Cumulative morphine consumption (mg)^a^	0 (0/0)	0 (0/0)	0.501

*VAS* visual analog scale

^a^Presented as median (1st and 3rd quartile)

## Discussion

Several clinical trials have studied the efficacy of palonosetron in the management of PONV, and almost all of them have reported a beneficial role of palonosetron when compared to placebo or other 5HT-3 receptor antagonists [8, 9, 15–18]. Two very recent meta-analyses showed reductions in the incidence of PONV when palonosetron was compared with ondansetron in patients who underwent general anesthesia [11, 12]. To our knowledge, the current study is the first to compare palonosetron with ondansetron in patients at a high risk of PONV who received neuraxial anesthesia with intrathecal morphine.

In the current study, there was no statistically significant reduction in the overall incidence of PONV in patients who received palonosetron versus those who received ondansetron, which suggests that the prophylactic effect of palonosetron on PONV is similar to that of other 5HT-3 receptor antagonists in patients undergoing neuraxial anesthesia. However, we observed a significant reduction in the incidence of late-onset vomiting with palonosetron, which could be attributable to the longer duration of action of palonosetron. Thus, palonosetron may better attenuate the prolonged adverse effects of intrathecal morphine than shorter acting 5HT-3 receptor antagonists.

Few studies have focused on PONV in patients undergoing regional anesthesia with neuraxial opioids [5]. In a recent meta-analysis, 5HT-3 receptor antagonists (not including palonosetron) were shown to significantly reduce the incidence of PONV after caesarean section performed under spinal anesthesia with intrathecal morphine [19]. In the only study evaluating the efficacy of palonosetron after spinal anesthesia, palonosetron was associated with a lower incidence of PONV than ramosetron, another long-acting 5HT-3 receptor antagonist [10]. That study was performed in patients who underwent caesarean section without receiving neuraxial opioids; thus, the incidence of PONV and other pathophysiological considerations in that study may not be comparable to those of the current study.

Nausea and vomiting are well-known side effects of opioids that may have different central and peripheral components. The precise mechanisms of opioid-induced nausea and vomiting (OINV) are not entirely certain; however, known aspects include the activation of mu opioid receptors in the chemoreceptor trigger zone, direct stimulation of the vestibular apparatus, and peripheral action of opioids on the gastrointestinal tract [20]. The use of morphine in neuraxial anesthesia is related to a high incidence of PONV. Any hydrophilic substance (e.g., morphine), when injected into the subarachnoid space, tends to remain in the cerebrospinal fluid for a long period; during this period, it moves rostrally and reaches areas in the brainstem that induce nausea and vomiting [5].

It is likely that the addition of morphine in neuraxial anesthesia contributed to the high incidence of nausea and vomiting observed in this study. However, because several mechanisms and risk factors were involved (e.g., young age, female sex, a history of nonsmoking, and a history of gynecological surgery), it is difficult to clearly separate “pure” OINV from the broader definition of PONV.

The underlying pathophysiological mechanisms of PONV after neuraxial anesthesia include the use of hydrophilic opioids, arterial hypotension, increased sensory blockade, symptomatic liquoric hypotension, and gastrointestinal hyperactivity due to sympathetic blockade [5]. Serotonin receptor antagonists may only counteract some of these factors. Therefore, other strategies for PONV prevention must be used in some cases.

Dexamethasone is an effective drug for PONV prophylaxis, including in patients who receive neuraxial opioids [21]. The combination of dexamethasone and ondansetron has been shown to be particularly effective in patients at high risk of PONV [22]. Moreover, it has recently been reported that the combination of dexamethasone and palonosetron is more effective than palonosetron alone for reducing PONV after laparoscopic surgery [23].

Considering the high incidence of PONV in our study population, we believed it was ethically appropriate to use a combination therapy for PONV prevention rather than a single drug [1]. As patients in both groups received dexamethasone for this purpose, the overall incidence of PONV was probably diminished, which may have increased the chance of a type II error and reduced the detection power of this study. A similar limitation is applicable to secondary outcomes, such as the incidence of clinically significant PONV as determined by the simplified PONV impact scale. Notably, the incidences of clinically significant PONV were low in both groups.

The long-acting effect of palonosetron seems to be useful when a hydrophilic opioid, such as morphine, is administered intrathecally. However, despite the pharmacological benefits of palonosetron, the incidence of PONV remained high in the current study. Further studies are necessary to evaluate the role of palonosetron in the prevention of PONV in patients who undergo neuraxial anesthesia, particularly when a neuraxial opioid is used. It is also important to study the role of palonosetron in association with other PONV prophylaxis strategies.

## Conclusion

In conclusion, palonosetron exhibited efficacy similar to that of ondansetron for reducing the overall incidence of PONV in patients who underwent TAH under spinal anesthesia with intrathecal morphine; however, palonosetron did reduce the incidence of late-onset vomiting better than ondansetron.

## Data Availability

The datasets used and/or analyzed during the current study are available from the corresponding author on reasonable request.

## Abbreviations

OINV: Opioid-induced nausea and vomiting
PONV: Postoperative nausea and vomiting
TAH: Total abdominal hysterectomy
VAS: Visual analogue scale
